# Characteristics of patients presenting post-suicide attempt to an Academic Medical Center Emergency Department in Lebanon

**DOI:** 10.1186/s12991-018-0191-5

**Published:** 2018-05-25

**Authors:** Imad El Majzoub, Christopher El Khuri, Karim Hajjar, Ralphe Bou Chebl, Farid Talih, Maha Makki, Aurelie Mailhac, Gilbert Abou Dagher

**Affiliations:** 1 Department of Emergency Medicine, American University of Beirut Medical Center, Beirut, Lebanon; 2 Department of Psychiatry, American University of Beirut Medical Center, Beirut, Lebanon; 3 Clinical Research Institute, American University of Beirut Medical Center, Beirut, Lebanon

**Keywords:** Emergency Department, Mental health, Overdose, Suicide

## Abstract

**Background:**

Emergency Department (ED) visits for suicide attempts have been described worldwide; however, the populations studied were predominantly Western European, North American, or East Asian. This study aims to describe the epidemiology of ED patients presenting post-suicide attempt to an academic medical center in Lebanon and to report on factors that affect ED disposition.

**Methods:**

A retrospective cohort study was conducted between 2009 and 2015. Patients of any age group were included if they had presented to the ED after a suicide attempt. Patients with unintentional self-harm were excluded. Descriptive analysis was performed on the demographics and characteristics of suicide attempts of the study population. A bivariate analysis to compare the two groups (hospitalized or discharged) was conducted using Student’s *t* test and Pearson Chi-square where appropriate. A multivariate analysis was then conducted to determine the predictors of hospital admission.

**Results:**

One hundred and eight patients were included in the final analysis. Most patients were females (71.4%) and between 22 and 49 years of age. A considerable number of patients were unemployed (43%), unmarried (61.1%), and living with family (86.9%). Most suicide attempts were performed at home (93.5%) and on a weekday (71.3%). The most common mechanisms of injury were overdose with prescription medications (61.3%), overdose with over-the-counter drugs (27.9%), and self-inflicted lacerations (10.1%). The classes of medication most commonly abused were benzodiazepines (39.3%) followed by acetaminophen (27.3%). A large portion of our patients were admitted (70.3%), with the majority going to the psychiatric ward (71.1%). Of note, a quarter (27.5%) of our patients left the ED against medical advice, with 23.5% of admitted patients leaving the hospital before completion of treatment. The main predictors of admission were found to be overdose on prescription medications OR 9.25 (2.12–40.42 CI95%).

**Conclusions:**

The characteristics of our suicide attempters mirror those of international and regional suicide attempters. Further work is required to quantify the effect of voluntary refusal of hospital treatment, the repercussions of family, and financial barriers to healthcare and suicide as a whole in our society.

**Electronic supplementary material:**

The online version of this article (10.1186/s12991-018-0191-5) contains supplementary material, which is available to authorized users.

## Background

Per the World Health Organization’s (WHO) World Health Statistics report, there are an estimated 800,000 suicide deaths yearly, worldwide [1]. In the United States (US), Emergency Departments (EDs) deal annually with 5 million psychiatric emergencies and approximately 590,000 visits for intentional self-harm [2]. In addition, regardless of the ED presenting chief complaint, studies note that 8% of all adult ED patients have had recent suicidal ideation [3, 4].

Unsuccessful suicide attempts account for 70% of all non-fatal self-inflicted injuries treated in the ED and are positive predictors of future completed suicide [2, 5]. In fact, Haukka et al. [6] mention that the single strongest factor that predicts suicide is a prior history of a suicide attempt. It is, therefore, important to better understand these patients, so as to tailor preventative efforts accordingly.

The ED is usually the first point of contact with mental health and medical care for suicidal patients [7, 8]. In 2003, a study by Gairin et al. [9] showed that 39% of suicide victims visited the ED at one point in the year before their death. The ED can thus allow an important access to mental health services and could play a vital role in suicide prevention [7, 10, 11]. Therefore, a better understanding of the use of services provided in EDs and the disposition status for self-harming patients is vital to implement effective suicide prevention programs.

Most epidemiologic studies have focused on completed rather than attempted suicide [12, 13]. Of the studies that examined attempted suicide, the majority have focused on certain age groups and mechanism of injury in the ED, with a few studies fully characterizing patients that attempt suicide [14–16]. These have noted a preponderance of females and teenagers amongst those presenting to the ED for attempted suicide, with poisoning being the most frequent method of suicide attempt and self-harm seen in the ED [17]. It was also observed that ED physicians tend to discharge such patients when certain conditions are met, mainly including the lack of an immediate risk of completing suicide and the availability of close follow-up [18].

Lebanese individuals often rank family first in terms of priority among all social institutions in their country. As such, in Lebanon, family functioning is connected to collectivism, where there is emphasis on collective rather than individual action or identity; even when it pertains to the perceived psychological well-being of its members [19]. Despite there being modest published data on suicide attempts in the Levant and Middle East region, there are no studies that examine this public health concern from an ED setting, in such a society, where mental illness is often a taboo [20–22].

To address this knowledge gap, we conducted a retrospective cohort study to explore the epidemiology and characteristics of patients presenting post-suicide attempt to the ED of a tertiary medical center in Lebanon and to examine the factors that determine hospital admission in this vulnerable population.

Our primary aim is to aid in better recognizing patients at risk of attempting suicide, and in addressing the factors that hinder in-hospital admission, with the ultimate goal of reducing the likelihood of attempting suicide.

## Methods

### Study design and setting

This was an institutional review board (IRB) approved (ER.GA.08), single-center, retrospective, cohort study conducted in an academic ED of a large tertiary care center, in Beirut, Lebanon. Patients that presented to the ED between December 2009 and December 2015 had their medical charts queried via the hospital’s electronic health record (EHR), and all clinical information was extracted from scanned charts and electronic laboratory reports by dedicated research fellows. Data collectors were not blinded to the study hypothesis. However, before the initiation of data collection, multiple meetings with the principle investigator were conducted to standardize the process.

### Patient selection

The patients’ ED encounters were filtered by an experienced data user using the hospital’s EHR via structured keyword searches and ICD-9 coding (*International Statistical Classification of Diseases and Related Health Problems)* for patients that presented to the ED after a suicide attempt [22]. Search terms included “suicide”, “suicidal”, “hanging”, “suffocation”, “submersion”, “fall”, “poison”, “toxic”, “injury”, “injuries”, “burn”, and “wound”. The encounters were then screened by research fellows to exclude all those found to have unintentional intoxication or injury. No age restrictions were applied.

### Other factors that were examined

Information was obtained regarding patients’ general demographics and baseline characteristics (including social history, underlying psychiatric disease, who escorted them to the ED and insurance status), patients disposition [insurance status, family barrier/resistance to admission and whether the patient left against medical advice (AMA) or not], the characteristics of the attempt (mechanism of injury, location of attempt, day of attempt, history of a previous attempt, time interval between attempts, and concomitant alcohol or drug abuse), and the characteristics of the hospital stay [hospital length of stay (LOS), intensive care unit (ICU) versus general medical floor admission, recovery status, financial and family barriers to hospital stay, in addition to leaving the hospital AMA].

Age was subdivided into three categories (≤ 21, 22–49, and ≥ 50) to normalize the distribution. Occupation status was reported as ‘employed’ if the patients had at least a part-time or a full-time job. Social history included occupation, living arrangement, marital status, and residence status.

Injury was considered intentional if the patients declared so themselves.

Mechanism of injury, location of attempt, day of attempt, history of previous attempt, time interval between attempts, and concomitant alcohol or drug abuse were also collected.

Hospital length of stay (LOS), intensive care unit (ICU) versus general medical floor admission, recovery status, financial and family barriers to hospital stay, and disposition status, in addition to leaving the hospital AMA were noted.

“Relationship or marriage problems” were noted from the assessment note of the treating physician if the patient expressed that his or her marriage or relationship was directly or indirectly associated with the suicide. “Underlying psychiatric disorder” was defined as the primary psychiatric disorder as indicated in the chart or as per diagnosis of treating psychiatrist. If the patient suffered from multiple psychiatric disorders as per the chart, the more severe disorder (disorder-specific rate of attempting suicide) was considered as the primary illness. “Family barrier to ED admission” and “Family barrier to hospital stay” were considered positive if any health care provider had clearly documented that the patient’s family contributed to the decisions of discharging patients from the ED and from the hospital, respectively. “Financial barrier to hospital stay” was considered positive if follow-up notes on the floors documented that the patient had financial issues impairing hospital stay. Finally, upon discharge from the hospital, patients were noted to have either “Complete recovery” or “Residual signs and symptoms” from their suicide attempt by examining pre-discharge assessments of the treating physician or on-call nurse.

### Statistical analysis

Statistical analyses were performed using SPSS version 24.0 (Armonk, NY: IBM Corp). The distributions of the continuous and categorical variables were presented as mean ± standard deviation and frequency/percentages, respectively. Selected characteristics were then stratified by admission status (discharged or admitted) and variable differences between the two groups were calculated by Pearson’s Chi-square/Fisher’s exact test and Student’s *t* test where appropriate. Tests were interpreted at a significance level alpha = 0.05. If a variable had missing data, percentages were calculated as per the remaining number of available data points.

A multivariate analysis was performed to ascertain the predictive factors of hospital admission in the population via logistic regression. A stepwise regression with an entry *p* value of 0.15 and a stay *p* value of 0.25 was conducted. The independent variables chosen for modeling were those found to be significant at the bivariate analysis level in addition to those considered clinically meaningful. The variables included in the model were: age, gender, marital status, employment status, underlying psychiatric disorder, mechanism used, history of previous attempt, insurance status, and psychiatric consultation in the ED. The results were described as odds ratios (OR) and their corresponding 95% confidence.

## Results

One hundred and eight patients were included in the final analysis, with most patients being females (71.4%) and between the ages of 22–49 years. A significant number of our patients were unemployed (43%), unmarried (61.1%), living with family (86.9%), and accompanied to the ED by family, as well (78.4%). One-third of the patients (35.8%) noted that their relationship or marriage was directly associated with their suicide attempt, with 10.5% noting that it contributed indirectly. The main underlying psychiatric disorder in our study population was depression (64.8%), while 23.1% of patients did not have any psychiatric illness prior to the suicide attempt. In terms of financial status, 65.7% were self-payers, with the rest being insured. In total, the treating physicians noted that 19.2% of patients had some form of family barrier and or resistance to hospital admission from the ED. Ninety-nine patient charts had documentation on whether or not the patient left AMA, of which a total of 27 patients (27.3%) left AMA, with 19 (19.2%) leaving AMA due to familial influence and 8 (8.1%) leaving as per the patients’ preference (Table 1).

**Table 1:** Demographics of patients presenting to the emergency department post-suicide attempt

(*N* = 108)	No. (%)
Male	32 (29.6)
Age in years	
≤ 21	28 (25.9)
22–49	60 (55.6)
≥ 50	20 (18.5)
≥ 65	4 (3.7)
Employment status^a^	
Employed	27 (29.0)
Unemployed	40 (43.0)
Student	26 (28.0)
Marital status	
Single^b^	66 (61.1)
Married	42 (38.9)
Relationship or marriage problems’ effect on the attempt^a^	
None	51 (53.7)
Direct	34 (35.8)
Indirect	10 (10.5)
Residential status^a^	
Family	93 (86.9)
Other^c^	14 (13.1)
Underlying psychiatric disorder	
Depression	70 (64.8)
None	25 (23.1)
Schizophrenia	8 (7.4)
Other^d^	5 (4.6)
Patient accompaniment^a^	
ED with family	80 (78.4)
ED with friends	7 (6.9)
ED with EMS	7 (6.9)
Other^e^	8 (7.8)
Insurance status^a^	
Self	65 (65.7)
Insured^f^	34 (34.3)
Family barrier to hospital admission	19 (19.2)
Left the ED AMA^a^	27 (27.3)
Yes, as per family	19 (19.2)
Yes, as per patient	8 (8.1)
No	72 (72.7)

*EMS* emergency medical services, *ED* Emergency Department, *AMA* against medical advice

^a^ Variables with missing values

^b^ Single includes divorced patients and those in relationships (not married)

^c^ 12 patients lived alone and 2 were domestic workers living at employer’s home

^d^ Included 2 with substance abuse disorder and 3 with general anxiety disorder

^e^ Includes 3 presenting with personal drivers, 3 with housekeepers, and 2 with strangers

^f^ Though many of our patients were insured, some insurance companies do not cover psychiatric-related hospitalization

Most attempts occurred at home (93.5%) and on a weekday (71.3%). The most common mechanisms used were overdose on prescription medications (pharmaceutical drugs requiring a medical prescription to be dispensed) (61.3%) followed by overdose on over-the-counter drugs (27.9%) and self-laceration (10.1%). 14.2 and 5.7% of patients presented with concomitant alcohol and illicit drug abuse, respectively. 38 patients (35.2%) had at least one prior suicide attempt, with most prior attempts occurring months (41.9%) or years (45.2%) before the current suicide attempt (Table 2).

**Table 2:** Characteristics of suicide attempts

(*N* = 108)	No. (%)
Mechanism used	
Prescription medications	65 (61.3)
Over-the-counter drugs	29 (27.9)
Self-laceration	11 (10.1)
Other^b^	7 (6.5)
Non-ingestible substances^c^	6 (5.5)
Location of attempt^a^	
Home	100 (93.5)
Other^d^	7 (6.5)
Day of the week attempt	
Week day	77 (71.3)
Weekend	31 (28.7)
Concomitant alcohol abuse	15 (14.2)
Concomitant drug abuse	6 (5.7)
Previous attempt	
At least one previous attempt	38 (35.2)
Time interval between two consecutive attempts^a^	
Days (< 7 days)	2 (6.5)
Weeks (≥ 7 days and < 4 weeks)	2 (6.5)
Months (≥ 4 weeks and < 12 months)	13 (41.9)
Years (≥ 12 months)	14 (45.2)

^a^ Variables with missing values

^b^ 3 attempted jumping from a lethal height, 1 firearm suicide, 1 attempted drowning, and 2 from pesticide ingestion

^c^ 2 attempts of swallowing bleach, 1 of soap, and 3 of unknown cleaning substances

^d^ 1 in a car, 1 at boyfriend’s house, 1 at sea, 1 at a hotel, and 3 at work)

84 out of the 108 patients had attempted suicide using at least one medication as their main attempt strategy, some of whom using multiple medications at the same time. The most commonly used medication in our study was found to be “Benzodiazepines” (39.3%), followed by “Acetaminophen” (27.3%). “Psychotropic drugs”, “Anti-depressants”, and “Cardiovascular medications” all had similar percentage of use at 17.5, 16.7, and 14.2%, respectively.

A large portion of our patients were admitted to the hospital (70.3%), with 54 (71.1%) going to the psychiatric ward, 18 (23.7%) to the ICU, and 4 (5.3%) to the general medical ward. The mean hospital length of stay was 3.43 ± 3.73 days. The treating physicians noted that 5.7 and 10.1% of admitted patients had a financial barrier and a family barrier to continued hospital stay, respectively. Eventually, among the patients who got admitted to the hospital, 10.3% left AMA with an additional 13.2% leaving AMA but at the request of the patient. In terms of outcome, three patients expired in-hospital (2.8%), 68 patients (63%) had full recovery, and more than a third (35.2%) suffered from residual signs and symptoms upon discharge. Of those that died, two were from polypharmacy prescription drugs and one due to fall from height. Of note, no patient presented dead on arrival.

Discharged patients were more likely to have used benzodiazepine as compared to admitted patients (43.8% vs 25.0%; *p* value = 0.05). Naturally, discharged patients had lower odds of being insured (21.9% vs 40.3%; *p* value = 0.07) as compared to being self-payers, and discharged patients had much higher odds of having family members oppose their admission from the ED (53.1% vs 3%; *p* value < 0.0001) (Table 3).

**Table 3:** Comparison between discharged and admitted post-suicide attempt patients

	Discharged *N* = 32	Admitted *N* = 76	*p* value
Male no. (%)	6 (18.8)	26 (34.2)	0.11
Age no. (%)			
≤ 21	4 (12.5)	24 (31.6)	0.06
22–49	23 (71.9)	37 (48.7)	
≥ 50	5 (15.6)	15 (19.7)	
Employment status no. (%)^a^			
Employed	7 (29.2)	20 (29.0)	0.62
Unemployed	12 (50.0)	28 (40.6)	
Student	5 (20.8)	21 (30.4)	
Marital status no. (%)^a^			
Single^b^	14 (48.3)	49 (64.5)	0.13
Married	15 (51.7)	27 (35.5)	
Residential status no. (%)^a^			
Family	28 (90.3)	65 (85.5)	0.75
Other^c^	3 (9.7)	11 (14.5)	
Underlying psychiatric disease no. (%)			
None	9 (28.1)	16 (21.1)	0.76
Schizophrenia	3 (9.4)	5 (6.6)	
Depression	19 (59.4)	51 (67.1)	
Other^d^	1 (3.1)	4 (5.3)	
Patient accompanied no. (%)^a^			
ED with family	23 (71.9)	57 (81.4)	0.45
ED with friends	4 (12.5)	3 (4.3)	
ED with EMS	2 (6.3)	5 (7.1)	
Other^e^	3 (9.4)	5 (7.1)	
Mechanism used no. (%)			
Over-the-counter drugs	11 (34.4)	18 (25.0)	0.32
Acetaminophen	7 (21.9)	16 (21.1)	0.92
Benzodiazepines	14 (43.8)	19 (25.0)	0.05
Prescription medications	18 (56.3)	47 (63.5)	0.48
Non-ingestible substances	2 (6.3)	4 (5.3)	1.00
Self-laceration	4 (12.5)	7 (9.2)	0.73
Other^f^	1 (3.1)	6 (7.9)	0.67
Venue of attempt no. (%)^a^			
Home	28 (87.5)	72 (96.0)	0.19
Other^g^	4 (12.5)	3 (4.0)	
Day of attempt no. (%)			
Week day	23 (71.9)	54 (71.1)	0.93
Weekend	9 (28.1)	22 (28.9)	
Concomitant alcohol abuse no. (%)	5 (15.6)	10 (13.5)	0.77
Concomitant drug abuse no. (%)	0 (0.0)	6 (8.1)	0.17
Previous attempt no. (%)			
At least one previous attempt	10 (31.1)	28 (36.8)	0.58
Time interval between two consecutive attempts no. (%)^a^			
Days	0 (0.0)	2 (8.7)	0.91
Weeks	1 (12.5)	1 (4.3)	
Months	3 (37.5)	10 (43.5)	
Years	4 (50.0)	10 (43.5)	
Insurance status no. (%)^a^			
Self	25 (78.1)	40 (59.7)	0.07
Insured^h^	7 (21.9)	27 (40.3)	
Family barrier for ED admission no. (%)	17 (53.1)	2 (3.0)	< 0.0001^a^
Psychiatry consulted in the ED no. (%)	25 (80.6)	43 (64.2)	0.10
Admission area			
Critical care	–	18 (23.7)	NA
Psychiatric ward	–	54 (71.1)	
Hospital floor	–	4 (5.3)	
Death			
Yes	2 (6.2)	1 (1.3)	0.21

*EMS* emergency medical services, *ED* Emergency Department

^a^ Variables with missing values

^b^ Single includes divorced patients and those in relationships (not married)

^c^ 12 patients lived alone and 2 were domestic workers

^d^ Included 2 with substance abuse disorder and 3 with general anxiety disorder

^e^ Includes 3 presenting with personal drivers, 3 with housekeepers, and 2 with strangers

^f^ 3 attempted jumping from a lethal height, 1 firearm suicide, 1 attempted drowning, and 2 from pesticide gas

^g^ 1 in a car, 1 at boyfriend’s house, 1 at sea, 1 at a hotel, and 3 at work (public building)

^h^ Though many of our patients were insured, some insurance companies do not cover psychiatric admissions. *p ≤ 0.05 considered significant

A multivariate analysis was performed to determine the predictors of admission considering all clinically relevant and statistically significant variables in the bivariate analysis (Additional file 1: Appendix S1, Additional file 2: Appendix S2). The main predictors of admission were found to be prescription medications overdose OR 9.25 (2.12–40.42 CI95%). Meanwhile, patients who were between 22 and 49 years of age, married, overdosed on benzodiazepines, and received psychiatric consultation in the ED were more likely to be discharged (Table 4).

**Table 4:** Multivariate regression: predictors of admission in patients presenting to the emergency department post-suicide attempt

Variables	OR (95% CI)
Age	
≤ 21	Reference
22–49 years	0.24 (0.08–0.69)
Marital status	
Single	Reference
Married	0.38 (0.13–1.14)
Medication used	
Prescription medicine	
No	Reference
Yes	9.25 (2.12–40.42)
Benzodiazepines	
No	Reference
Yes	0.10 (0.02–0.41)
At least one previous attempt	
No	Reference
Yes	2.38 (0.82–6.90)
Psychiatric consultation in the ED	
No	Reference
Yes	0.14 (0.04–0.52)

## Discussion

Worldwide, and in the Middle East, psych-related ED visits and suicide attempts continue to grow [14–16, 20, 21, 23]. Studies have shown that 1 in 4 Lebanese will suffer from mental illness in their lifetime [24]. In addition, mental health has long been an issue pervaded by stigma in Lebanon. It is, therefore, vital that this vulnerable population be studied in the unique ED setting to better characterize the presentations and optimize the acute care of suicidal patients. Our study looked at 108 patients that presented to the ED of a tertiary care medical center in Beirut, Lebanon for a suicide attempt of any mechanism.

The majority of our patients were female, between 22 and 49 years of age, unemployed, single, living with family, and suffering from depression. Their attempts were carried out predominantly at home, on a weekday, and via overdose on prescription drugs. Data from the World Bank in Lebanon revealed that unemployment rates in 2016 were higher in females (11% of female labor force) as compared to males (5.4% of male labor force) [25]. This may justify why the majority of the patients enrolled in our study were females and unemployed. The fact that the attempts were carried out predominantly at home on a weekday could partially reflect ‘ideal circumstances’ for a suicide attempt; during a time at which the male co-habitants are conveniently busy with their jobs.

Data from China [15], Brazil [23], and Japan [26] also show a predominance of females in ED visits for suicide attempts, with percentages similar to ours. In the literature, females have been found to attempt more suicides, while men tend to more successfully complete their attempts [27]. This may explain our female predominance considering that our patients are those that presented to the ED after an incomplete suicide attempt.

In a study by Oliffe et al. [28], a greater percentage of male respondents in Canada expressed that they would feel embarrassed to seek help for depression.

Moreover, many argue that, in Lebanon, men have a superior status in society and that many Lebanese family structures are patriarchal. Seeking help for mental illness may be perceived as a sign of weakness, in a society where men are often perceived as authoritative figures. In fact, Karam et al. [29] showed that older Lebanese women were 3.1 times more likely to report a 12-month mental health disorder, as compared to older men.

Our population was notably young with more than 50% between the ages of 22 and 49 years. Caterino et al. [2] noted in their large US-based cohort on suicidal patients that 84% of them were below the age of 65 years. Likewise, Zhao et al. [15] in China showed that 52.9% of those presenting after a suicide attempt were between 21 and 30 years of age. Alves et al. [23] echoed this a predominance of ED patients between the ages of 20–29 years in Brazil. A possible reason behind why the young present more for suicide attempts may stem from job and relationship related stressors, both abundant in the formative years of young adults [23–26]. Karam et al. [29] also showed that, in Lebanon, adults aged 60 years and above had a significantly lower prevalence of mental health disorders as compared to those younger than 60. Similarly, there is recent evidence to suggest that suicide rates in the United States appear to be increasing among middle-aged adults and that rates for adults ≥ 65 years of age appear to be decreasing [30]. This difference could be due to poorer recall of past episodes, better coping skills, and greater stigma towards seeking help for mental illness in the elderly [31–33]. Another explanation would be the non-availability of elderly due to mortality or illness which may lead to a sampling bias [32].

Focusing on medication-based suicides, the previous studies have attempted to characterize the medication profile of suicide attempts by looking at acute medication overdose visits to the ED. In the Middle East, a study by Bakhaidar et al. [21] showed that analgesics and non-steroidal anti-inflammatory drugs (NSAIDs) were the most commonly used medications due to ease of procurement in their Saudi-based population. A study by Zohre et al. [20] in Iran showed that the most common single drugs abused were also analgesics (without acetaminophen) in addition to anti-depressants. In Italy, Zeppegno et al. [14] showed that benzodiazepines and barbiturates were the most commonly used drugs for overdose and a Korean study by Jang et al. [34] also showed that psychotropic drug overdose were the most utilized among Koreans. In the US, Ting et al. [17] showed that unspecified drugs (mainly over-the-counter medications) were the most common method to overdose, followed by with tranquilizers and psychotropics. In our study, benzodiazepines were the most commonly used method to overdose, which was similar to the finding of the Italian study. Although most countries were found to have predominantly medication-based suicide attempts, the medication profiles of these attempts differed in every country. We believe that the predominance of benzodiazepine use in our population stems from our physicians’ lower threshold for prescribing these medications and a chronic oversight and diversion control problems in pharmacies, leading to an increased availability of these drugs to the public [35].

Our multivariate analysis showed prescription medication overdose to be a strong predictor of admission after a suicide attempt. Prescription medications including cardiovascular and psychotropic drugs, in overdoses, may cause severe toxidromes requiring hospital admission and treatment. Though certain over-the-counter medication overdoses such as aspirin, NSAIDs or acetaminophen can also lead to severe toxidromes, we did not find them predictors of hospital admission. This may be due to their underrepresentation in our study as only a few patients presented with aspirin or NSAID overdose and most acetaminophen intoxications were moderate in severity. History of at least one previous attempt was also a predictor of hospital admission but not found to be statistically significant. In the literature, the previous attempts are considered major risk factors for suicide attempts as per the widely used SAD PERSONS psychometric scale [36]. Suicidal patients with the previous suicide attempts are at a higher risk of repeating and completing the attempt and thus would require hospital admission.

“Emergency commitment”, i.e., the involuntary admission of suicidal patients, is an important intervention aimed to protect suicidal patients presenting to the ED [3]. Unlike the US, Lebanon does not have any specific medico-legal framework to deal with psychiatric emergency cases, including suicidality. In addition, as our study shows, leaving the hospital AMA is highly dependent on the patient and often on the family’s attitude towards mental illness. Over a quarter of our patients left the ED AMA with an almost equal proportion leaving the hospital before completion of treatment. In our study, 19.2% of patients had some form of family barrier to hospital admission and 10.1% to hospital stay. In fact, the bivariate analysis showed that 53.1% of discharged patients had a family barrier to hospital admission. Suicide is considered a taboo in Lebanon and the surrounding region due to social and religious reasons [20, 21]. Due to this stigma, many families and patients prefer to continue treatment out of hospital, in the privacy of their own homes. In addition to family barriers, most of our patients were uninsured (65.7%) and even those insured had very limited to no mental health coverage included in their insurance policies. This is due to a long-standing deficit in mental health insurance coverage by both private and public parties in Lebanon. It was not until April 2017 that the Lebanese ministry of public health declared an official plan to cover patients for mental illness. Certain developed countries, such as Switzerland and the United States, only recently passed laws or revisited them (in 2008) requiring private and public insurance parties to cover mental illnesses. Meanwhile, countries such as Canada and the United Kingdom have mental health coverage included in their universal care systems [37]. A large community survey by Strum et al. [38] showed that the absence of such universal mental health coverage is a major deterrent for seeking treatment. In Lebanon, not only is this apparent (our sample size may have been affected by this) but even some of those that reach the hospital, leave AMA due to financial constraints. Both family and financial barriers lead to incomplete care which may have a negative impact on patients and predispose them to repeated suicide attempts. More work is required to further quantify the effect of voluntary refusal of hospital treatment for suicide patients in the acute setting.

Having a history of a suicide attempt causes stigmatizing and attitudinal barriers to seeking help. Prevention strategies ought to consequently target individuals with a suicidal past with special consideration for self-stigma and feelings of indignity that come with help-seeking [39].

We suggest the implementation of a thorough suicide screening protocol, with more scrutiny of patients with the following characteristics: females, age between 20 and 50 years, unemployed, unmarried, having marital or relationship problems, having a psychiatric illness and having access to psychotropic medications.

Another potentially effective intervention is instituting a more stringent medication-refill protocol (e.g., benzodiazepines), which allows prescribers to periodically re-evaluate their patients, to effectively screen for suicidal ideations and prevent accordingly.

We also suggest addressing the barriers towards hospital admission, notably the financial and family barriers. We believe that addressing those barriers can have the most impact in patients with red flags that suggest ensuing suicidality, such as those with a plan, access to firearms, and those lacking social support.

The financial barriers can be addressed by the establishment and support of non-governmental organizations (NGOs) that provide financial assistance to patients who cannot afford mental health treatment, and by nationally lobbying with the insurance companies to cover psychiatric admissions, which they do not currently do. Most importantly, awareness should be raised around mental health in Lebanon and the Middle East by dispelling the myths that surround mental illness. This will hopefully legitimize mental illness as a serious public health concern, and will be a step closer towards having third-party payers cover for mental health treatment expenses. NGOs can also be critical in targeting family barriers to admission, notably by raising awareness about the importance of seeking help for mental illness and by reducing the stigma that comes with it.

## Limitations

In terms of number of patients, the authors acknowledge that 108 attempted suicide cases in a 6-year period are limited. From a methodology standpoint, patients that attempted suicide and presented to our ED may sometimes be coded via ICD-9 as something not related to our keyword searches. Beyond the study’s methodology, our patient sample may have been affected by the stigma regarding mental illness in our local society that discourages seeking medical attention after a suicide attempt. Moreover, until very recently, the ED was mandated to report suicide attempts to the police, who would then launch an investigation. This may have led physicians to under report suicide attempts to avoid the hassle of having to deal with the police. In addition, as per anecdotal law enforcement reports and preliminary fieldwork, it is estimated that one-third of suicides are underreported via the generic diagnosing of “accidental death” due to family preferences and often religious reasons, as religious burial rites are sometimes withheld from those who die by suicide in Lebanese culture.

In terms of study methodology, our study is subject to some biases. First, the setting of the study could only be conducted in our single urban tertiary hospital’s ED in the relatively more affluent capital city due to logistical reasons. Patients that present to our ED are on average more capable financially and more educated, possibly limiting the generalizability of our results to the general national population. We could also identify a few sources of information bias and measurement errors. In fact, information from other hospitals, which could have possibly provided information regarding the previous suicide attempts, could not be retrieved. Moreover, some of our study variables such as “underlying psychiatric disease”, “family barrier to admission”, and “relationship or marriage problems” may not have been measured and or reported in a standardized way; instead, they were inferred by the notes that health care professionals wrote during the ED visit. Finally, in the multivariate analysis, insurance status and family barriers did not appear in the model. In terms of insurance status, it was included in the analysis, however, which was removed from the model, because many patients with financial constraints were still admitted to the hospital. However, these admitted patients would be discharged a day or 2 afterwards because of inability to continue payment. Though this would manifest as no difference in financial barriers to hospital admission between admitted vs discharged patients, it would still signal the effect of financial barriers on completion of care. As for family barriers, it was not included in the analysis because of a statistical issue (low sample size) during modeling. Only 2 (3%) patients had a family barrier and were subsequently admitted vs 17 (53.1%) (*p* ≤ 0.001) that were discharged (Table 4). Though not included in the analysis, the authors note that this discrepancy is a reflection of the hindering effect of family resistance on hospital admission and the receiving of adequate treatment in this vulnerable population.

## Conclusions

This study aims to add to the literature of suicide attempters presenting to the ED and sheds light on their demographics, previous psychiatric history, and detailed mechanism of suicide along with hospital disposition. The characteristics of our suicide population treated in an urban ED in Lebanon mirror those of international and regional suicide patients. Benzodiazepines were the most frequent drugs ingested to attempt suicide and their use was protective against hospital admission from the ED. Institution of a law that mandates stringent refill protocols is therefore necessary. Further work is required to assess suicide in our country and region, as well as quantify the effect of voluntary refusal of hospital treatment, the repercussions of family interference, and the financial barriers to healthcare on the management of the suicidal patient. In addition, more public health initiatives are required to destigmatize mental illness, in general, and suicide, in particular, and to facilitate the acute medical and psychiatric treatment of this high-risk group of patients.

## Additional files

**Additional file 1: Appendix S1.** Sample of ICD-9 codes of patients presenting with self-harm used in our study.

**Additional file 2: Appendix S2.** Hospital course (N = 76).

## Abbreviations

WHO: World Health Organization
ED: Emergency Department
AMA: against medical advice
ICU: intensive care unit
OR: odds ratio
EMS: emergency medical services
NSAIDS: non-steroidal anti-inflammatory drugs
