# Supplementary material for: Characteristics of patients presenting post-suicide attempt to an Academic Medical Center Emergency Department in Lebanon
Source: Ann Gen Psychiatry. 2018 May 25;17:21. doi: 10.1186/s12991-018-0191-5 (PMC5970493; doi:10.1186/s12991-018-0191-5)
Supplement: Supplementary file 1 — Additional file 1: Appendix S1. Sample of ICD-9 codes of patients presenting with self-harm used in our study. [file 12991_2018_191_MOESM1_ESM.docx]

**Appendix S1: Sample of ICD-9 codes of patients presenting with self-harm used in our study**

| 900 | Injury to blood vessels of head and neck |
| --- | --- |
| 901 | Injury to blood vessels of thorax |
| 902 | Injury to blood vessels of abdomen and pelvis |
| 903 | Injury to blood vessels of upper extremity |
| 904 | Injury to blood vessels of lower extremity and unspecified sites |
| 905 | Late effects of musculoskeletal and connective tissue injuries |
| 906 | Late effects of injuries to skin and subcutaneous tissues |
| 907 | Late effects of injuries to the nervous system |
| 908 | Late effects of other and unspecified injuries |
| 909 | Late effects of other and unspecified external causes |
| 910 | Superficial injury of face neck and scalp except eye |
| 911 | Superficial injury of trunk |
| 912 | Superficial injury of shoulder and upper arm |
| 913 | Superficial injury of elbow forearm and wrist |
| 914 | Superficial injury of hand(s) except finger(s) alone |
| 915 | Superficial injury of finger(s) |
| 916 | Superficial injury of hip thigh leg and ankle |
| 917 | Superficial injury of foot and toe(s) |
| 918 | Superficial injury of eye and adnexa |
| 919 | Superficial injury of other multiple and unspecified sites |
| 940 | Burn confined to eye and adnexa |
| 941 | Burn of face head and neck |
| 942 | Burn of trunk |
| 943 | Burn of upper limb except wrist and hand |
| 944 | Burn of wrist(s) and hand(s) |
| 945 | Burn of lower limb(s) |
| 946 | Burns of multiple specified sites |
| 947 | Burn of internal organs |
| 948 | Burns classified according to extent of body surface involved |
| 949 | Burn unspecified site |
| 960 | Poisoning by antibiotics |
| 961 | Poisoning by other anti-infectives |
| 962 | Poisoning by hormones and synthetic substitutes |
| 963 | Poisoning by primarily systemic agents |
| 964 | Poisoning by agents primarily affecting blood constituents |
| 965 | Poisoning by analgesics antipyretics and antirheumatics |
| 966 | Poisoning by anticonvulsants and anti-parkinsonism drugs |
| 967 | Poisoning by sedatives and hypnotics |
| 968 | Poisoning by other central nervous system depressants and anesthetics |
| 969 | Poisoning by psychotropic agents |
| 970 | Poisoning by central nervous system stimulants |
| 971 | Poisoning by drugs primarily affecting the autonomic nervous system |
| 972 | Poisoning by agents primarily affecting the cardiovascular system |
| 973 | Poisoning by agents primarily affecting the gastrointestinal system |
| 974 | Poisoning by water mineral and uric acid metabolism drugs |
| 975 | Poisoning by agents primarily acting on the smooth and skeletal muscles and respiratory system |
| 976 | Poisoning by agents primarily affecting skin and mucous membrane ophthalmological otorhinolaryngological and dental drugs |
| 977 | Poisoning by other and unspecified drugs and medicinal substances |
| 978 | Poisoning by bacterial vaccines |
| 979 | Poisoning by other vaccines and biological substances |
| E950.0 | Suicide and self-inflicted poisoning by analgesics, antipyretics, and antirheumatics |
| E950.1 | Suicide and self-inflicted poisoning by barbiturates |
| E950.2 | Suicide and self-inflicted poisoning by other sedatives and hypnotics |
| E9503 | Suicide and self-inflicted poisoning by tranquilizers and other psychotropic agents |
| E9504 | Suicide and self-inflicted poisoning by other specified drugs and medicinal substances |
| E9505 | Suicide and self-inflicted poisoning by unspecified drug or medicinal substance |
| E9506 | Suicide and self-inflicted poisoning by agricultural and horticultural chemical and pharmaceutical preparations other than plant foods and fertilizers |
| E9507 | Suicide and self-inflicted poisoning by corrosive and caustic substances |
| E9508 | Suicide and self-inflicted poisoning by arsenic and its compounds |
| E9509 | Suicide and self-inflicted poisoning by other and unspecified solid and liquid substances |
| E9510 | Suicide and self-inflicted poisoning by gas distributed by pipeline |
| E9511 | Suicide and self-inflicted poisoning by liquefied petroleum gas distributed in mobile containers |
| E9518 | Suicide and self-inflicted poisoning by other utility gas |
| E9520 | Suicide and self-inflicted poisoning by motor vehicle exhaust gas |
| E9521 | Suicide and self-inflicted poisoning by other carbon monoxide |
| E9528 | Suicide and self-inflicted poisoning by other specified gases and vapors |
| E9529 | Suicide and self-inflicted poisoning by unspecified gases and vapors |
| E9530 | Suicide and self-inflicted injury by hanging |
| E9531 | Suicide and self-inflicted injury by suffocation by plastic bag |
| E9538 | Suicide and self-inflicted injury by other specified means |
| E9539 | Suicide and self-inflicted injury by unspecified means |
| E954 | Suicide and self-inflicted injury by submersion [drowning] |
| E9550 | Suicide and self-inflicted injury by handgun |
| E9551 | Suicide and self-inflicted injury by shotgun |
| E9552 | Suicide and self-inflicted injury by hunting rifle |
| E9553 | Suicide and self-inflicted injury by military firearms |
| E9554 | Suicide and self-inflicted injury by other and unspecified firearm |
| E9555 | Suicide and self-inflicted injury by explosives |
| E9556 | Suicide and self-inflicted injury by air gun |
| E9557 | Suicide and self-inflicted injury by paintball gun |
| E9559 | Suicide and self-inflicted injury by firearms and explosives, unspecified |
| E956 | Suicide and self-inflicted injury by cutting and piercing instrument |
| E9570 | Suicide and self-inflicted injuries by jumping from residential premises |
| E9571 | Suicide and self-inflicted injuries by jumping from other man-made structures |
| E9572 | Suicide and self-inflicted injuries by jumping from natural sites |
| E9579 | Suicide and self-inflicted injuries by jumping from unspecified site |
| E9580 | Suicide and self-inflicted injury by jumping or lying before moving object |
| E9581 | Suicide and self-inflicted injury by burns, fire |
| E9582 | Suicide and self-inflicted injury by scald |
| E9583 | Suicide and self-inflicted injury by extremes of cold |
| E9584 | Suicide and self-inflicted injury by electrocution |
| E9585 | Suicide and self-inflicted injury by crashing of motor vehicle |
| E9586 | Suicide and self-inflicted injury by crashing of aircraft |
| E9587 | Suicide and self-inflicted injury by caustic substances, except poisoning |
| E9588 | Suicide and self-inflicted injury by other specified means |
| E9589 | Suicide and self-inflicted injury by unspecified means |
| E959 | Late effects of self-inflicted injury |
| E9808 | Poisoning by arsenic and its compounds, undetermined whether accidentally or purposely inflicted |
| E9809 | Poisoning by other and unspecified solid and liquid substances, undetermined whether accidentally or purposely inflicted |
| E9810 | Poisoning by gas distributed by pipeline, undetermined whether accidentally or purposely inflicted |
| E9811 | Poisoning by liquefied petroleum gas distributed in mobile containers, undetermined whether accidentally or purposely inflicted |
| E9818 | Poisoning by other utility gas, undetermined whether accidentally or purposely inflicted |
| E9820 | Poisoning by motor vehicle exhaust gas, undetermined whether accidentally or purposely inflicted |
| E9821 | Poisoning by other carbon monoxide, undetermined whether accidentally or purposely inflicted |
| E9828 | Poisoning by other specified gases and vapors, undetermined whether accidentally or purposely inflicted |
| E9829 | Poisoning by unspecified gases and vapors, undetermined whether accidentally or purposely inflicted |
| E9830 | Hanging, undetermined whether accidentally or purposely inflicted |
| E9831 | Suffocation by plastic bag, undetermined whether accidentally or purposely inflicted |
| E9838 | Strangulation or suffocation by other specified means, undetermined whether accidentally or purposely inflicted |
| E9839 | Strangulation or suffocation by unspecified means, undetermined whether accidentally or purposely inflicted |
| E984 | Submersion (drowning), undetermined whether accidentally or purposely inflicted |
| E9850 | Injury by handgun, undetermined whether accidentally or purposely inflicted |
| E9851 | Injury by shotgun, undetermined whether accidentally or purposely inflicted |
| E9852 | Injury by hunting rifle, undetermined whether accidentally or purposely inflicted |
| E9853 | Injury by military firearms, undetermined whether accidentally or purposely inflicted |
| E9854 | Injury by other and unspecified firearm, undetermined whether accidentally or purposely inflicted |
| E9855 | Injury by explosives, undetermined whether accidentally or purposely inflicted |
| E9856 | Injury by air gun, undetermined whether accidental or purposely inflicted |
| E9857 | Injury by paintball gun, undetermined whether accidental or purposely inflicted |
| E986 | Injury by cutting and piercing instruments, undetermined whether accidentally or purposely inflicted |
| E9870 | Falling from residential premises, undetermined whether accidentally or purposely inflicted |
| E9871 | Falling from other man-made structures, undetermined whether accidentally or purposely inflicted |
| E9872 | Falling from natural sites, undetermined whether accidentally or purposely inflicted |
| E9879 | Falling from unspecified site, undetermined whether accidentally or purposely inflicted |
| E9880 | Injury by jumping or lying before moving object, undetermined whether accidentally or purposely inflicted |
| E9881 | Injury by burns or fire, undetermined whether accidentally or purposely inflicted |
| E9882 | Injury by scald, undetermined whether accidentally or purposely inflicted |
| E9883 | Injury by extremes of cold, undetermined whether accidentally or purposely inflicted |
| E9884 | Injury by electrocution, undetermined whether accidentally or purposely inflicted |
| E9885 | Injury by crashing of motor vehicle, undetermined whether accidentally or purposely inflicted |
| E9886 | Injury by crashing of aircraft, undetermined whether accidentally or purposely inflicted |
| E9887 | Injury by caustic substances, except poisoning, undetermined whether accidentally or purposely inflicted |
| E9888 | Injury by other specified means, undetermined whether accidentally or purposely inflicted |
| E9889 | Injury by unspecified means, undetermined whether accidentally or purposely inflicted |
| E989 | Late effects of injury, undetermined whether accidentally or purposely inflicted |
