# Supplementary material for: Characteristics of patients presenting post-suicide attempt to an Academic Medical Center Emergency Department in Lebanon
Source: Ann Gen Psychiatry. 2018 May 25;17:21. doi: 10.1186/s12991-018-0191-5 (PMC5970493; doi:10.1186/s12991-018-0191-5)
Supplement: Supplementary file 2 — Additional file 2: Appendix S2. Hospital course (N = 76). [file 12991_2018_191_MOESM2_ESM.docx]

| Appendix S2: Hospital Course (N=76)^1^ | |
| --- | --- |
| Disposition no.(%)  Psychiatric ward  Critical care  General medical ward | 54 (71.1)  18 (23.7)  4 (5.3) |
| Hospital length of stay in days (Mean ± SD) | 3.43 ± 3.73 |
| Hospital mortality no.(%)^2^ | 3 (2.8) |
| Recovery status no.(%)  Complete  Residual signs and symptoms | 68 (63.0)  38 (35.2) |
| Financial barrier for hospital stay no.(%) | 4 (5.7) |
| Family barrier for hospital stay no.(%) | 7 (10.1) |
| Patient Left the hospital AMA no.(%)  Yes  Yes, as per patient  No | 7 (10.3)  9 (13.2)  52 (76.5) |
| ^1^2 patients were transferred to another hospital. 30 patients left the ED AMA.  ^2^Percentage done for N=108 total population. | |
